# A pilot study of the immunological profile and efficacy of rituximab in muscle-specific kinase antibody-positive myasthenia gravis

**DOI:** 10.3389/fimmu.2025.1624038

**Published:** 2025-07-25

**Authors:** Fengzhan Li, Zhouao Zhang, Tianyu Ma, Yuting Li, Luyao Su, Zhouyi Wang, Tiancheng Luo, Deyou Peng, Xinyan Guo, Mingjin Yang, Xue Du, Xiaoyu Huang, Yong Zhang

**Affiliations:** ^1^ People’s Hospital of Jiawang of Xuzhou, Jiawang Branch of Xuzhou Medical University Affiliated Hospital, Xuzhou, Jiangsu, China; ^2^ Department of Neurology, The Affiliated Hospital of Xuzhou Medical University, Xuzhou, Jiangsu, China

**Keywords:** muscle-specific kinase, myasthenia gravis, B lymphocyte, T lymphocyte, natural killer cell, rituximab

## Abstract

**Purpose:**

This study summarized the clinical and immunological characteristics of patients with muscle-specific kinase (MuSK) antibody-positive myasthenia gravis (MG), compared their difference with acetylcholine receptor (AChR) antibody-positive MG, and evaluated the therapeutic efficacy of rituximab (RTX) in MuSK-MG.

**Methods:**

This study included 10 MuSK-MG patients and 10 new-onset AChR-MG patients. Clinical and immunological data were collected from medical records before RTX treatment. The efficacy of RTX in MuSK-MG was evaluated by MG-specific activities of daily living (MG-ADL) and quantitative MG (QMG) scores.

**Results:**

All 10 MuSK-MG patients were female with a mean onset age of 44.3 ± 12.0 years, predominantly presenting with bulbar muscle weakness (90%) and limb weakness (80%). Compared to AChR-MG, MuSK-MG showed higher MG-ADL and QMG scores (P < 0.05), along with more frequent bulbar involvement at disease onset (P = 0.036). Immunological analyses revealed elevated CD19^+^B cells and memory B cells in MuSK-MG (P < 0.05). CD4^+^T cells and CD19^+^B cells showed positive correlations with QMG score (r = 0.766, P = 0.027; r = 0.767, P = 0.026), while natural killer (NK) cells were negatively correlated (r = -0.803, P = 0.005) in MuSK-MG. MuSK-MG patients had a mean MG-ADL score of 8.7 ± 2.5 at baseline. Following RTX treatment, MG-ADL score showed significant improvement, decreasing by -5.1 (95% CI: -7.6 to -2.6) at month 1 and -8.0 (95% CI: -11.0 to -5.0) at month 24. Nine patients took prednisone before RTX, with a median daily dosage of 40.0 mg, which decreased to 2.5 mg/day at month 6, and 8 of 9 (88.7%) patients discontinuing prednisone since month 12.

**Conclusion:**

MuSK-MG showed distinct clinical and immunological features, including predominant bulbar/limb onset, elevated CD19^+^B and memory B cells, and disease severity associated CD4^+^T, CD19^+^B and NK-cell alterations. In patients with MuSK-MG, low-dose RTX may be associated with long-term and sustained clinical improvement.

## Introduction

Myasthenia gravis (MG) represents a chronic autoimmune disorder mediated by pathogenic autoantibodies targeting components of the neuromuscular junction (NMJ), leading to characteristic clinical manifestations of skeletal muscle weakness and fatigability (1). Studies reported that approximately 80% of MG patients demonstrate detectable autoantibodies targeting the acetylcholine receptor (AChR), while the prevalence of muscle-specific tyrosine kinase (MuSK) antibodies only ranges from 1% to 10% (2). In addition, anti-lipoprotein-receptor-related protein 4 (LRP4) antibody is another type that contributes to MG and about 5% of patients remain seronegative (2). Anti-AChR antibodies exert their pathogenic effects through multiple mechanisms: (1) direct competitive inhibition of ACh binding to its receptor, (2) induction of AChR internalization and subsequent lysosomal degradation, and (3) activation of the complement cascade culminating in membrane attack complex (MAC) formation, collectively contributing to structural and functional impairment of the postsynaptic membrane at the NMJ (2). In contrast to AChR, MuSK is a single-pass transmembrane receptor tyrosine kinase that undergoes phosphorylation upon activation by the LRP4-agrin complex, a critical process for AChR clustering at the NMJ (3). Since the majority of anti-MuSK antibodies are classified as immunoglobulin G4 (IgG4), they interfere with LRP4 and other proteins such as collagen Q to inhibit the clustering of AChR, rather than activate the complement or induce antigenic modulation like anti-AChR antibodies (4).

The prevalence of MuSK-MG exhibits significant geographical and ethnic variations, demonstrating a consistent female predominance across different populations (5). MuSK-MG usually occurs in adults and is rare in children and those aged over 70 years old (6). In addition, MuSK-MG is not associated with thymic pathology, and clinical evidence suggests that thymectomy demonstrates limited therapeutic efficacy in MuSK-MG patients (7). MuSK-MG typically presents with an acute onset, frequently manifesting onset symptoms characterized by predominant bulbar muscle involvement (7). Furthermore, MuSK-MG exhibits unique clinical features including axial muscle weakness, presenting as head drop, accompanied by significant muscle atrophy particularly in facial muscles and the tongue (8–10). Although acetylcholinesterase inhibitors (AChEIs) have been traditionally used as first-line therapy for MG, a recent clinical investigation revealed that only 4.2% of MuSK-MG patients achieved initial clinical improvement with AChEI, while a substantial proportion (76.9%) experienced adverse effects, predominantly including neuromuscular hyperexcitability, gastrointestinal disturbances, and neurovegetative dysfunction (11). Furthermore, although corticosteroids are efficacy in MuSK-MG patients, it typically requires a higher dosage, potentially leading to significant long-term adverse effects and complications (12). Traditional immunosuppressants, including tacrolimus, azathioprine, and mycophenolate mofetil are also effectively administered alone or in combination with steroids in patients with MuSK-MG (5, 6). However, compared with AChR-MG, frequent relapses occur more frequently in those patients during the reduction of GC or non-steroidal immunosuppressant dosages (13).

Theoretically, as AChR antibodies are mainly IgG1 to IgG3 (produced by long-lived plasma cells that do not express CD20), while the main subtype of MuSK antibodies is IgG4, which is generated by short-lived plasmablasts, rituximab (RTX), a chimeric anti-CD20 monoclonal antigen, has no impact on long-lived plasma cells and demonstrates a better therapeutic effect on patients with MuSK-MG (14, 15). A meta-analysis including 99 patients with AChR-MG and 57 patients with MuSK-MG treated with RTX demonstrated that minimal manifestations (MM) or better were achieved in 72% of MuSK-MG patients compared to 30% in AChR-MG patients (16). Besides, MuSK-MG patients exhibited a significantly lower relapse rate following RTX treatment (16). Furthermore, cumulative evidence from multiple clinical studies has consistently shown both the efficacy and safety of RTX in the treatment of MuSK-MG, which has led to the growing recognition of RTX as a feasible therapeutic option in the early treatment stage following an inadequate response to conventional first-line therapies (13, 17–19). In addition, the effectiveness of other therapies, including interleukin-6 (IL-6) inhibitors, monoclonal antibodies (anti-CD19, anti-CD38, and anti-CD40), and chimeric autoantibody receptor T cells still need more clinical research to verify.

In this retrospective cohort study, we analyzed the clinical and immunological features of 10 patients diagnosed with MuSK-MG who were treated in our department. The objective of this investigation was to delineate the distinctive clinical and immunological characteristics of MuSK-MG and to observe the efficacy of RTX in MuSK-MG.

## Materials and methods

### Subjects

This retrospective study included a total of 10 MuSK-MG patients and 10 age- and gender-matched new-onset AChR-MG patients admitted to the Department of Neurology at the Affiliated Hospital of Xuzhou Medical University between July 2021 and February 2024. The diagnosis of MG was based on the presence of typical symptoms, such as fluctuating skeletal muscle weakness, and at least one of the following criteria: (a) positivity for anti-AChR or anti-MuSK antibodies; (b) repetitive nerve stimulation (RNS) at a frequency of 3 Hz shows a decrement of > 10% from the first to the fourth compound muscle action potential (CMAP); (c) positive neostigmine test. Patients were excluded if they met any of the following criteria: (a) incomplete medical records; (b) a history of infection within the past 3 months; or (c) severe cardiac, hepatic, or renal dysfunction, other autoimmune diseases, or malignant tumors (except thymoma).

### Data collection and follow-up

Demographic data, including age, gender, disease duration, symptoms at disease onset, and previous treatments, were collected from medical records. The baseline disease activity was evaluated using the Myasthenia Gravis Foundation of America (MGFA) classification, quantitative MG (QMG) score, and MG activities of daily living (MG-ADL) score before RTX treatment. QMG score was evaluated more than 8 hours after the last use of pyridostigmine. Venous blood samples were obtained in the early morning within 24 hours of admission and were obtained before treated with RTX in patients with MuSK-MG. Peripheral T lymphocytes subset (including CD3^+^T cells, CD4^+^T cells, and CD8^+^T cells), B lymphocytes subset (including CD19^+^B cells, memory B cells, plasmablasts, and plasma cells), and natural killer (NK) cells were measured by flow cytometry (BD LSRF Ortessa, Franklin Lakes, NJ, USA). Memory B cells were defined as CD45^+^CD19^+^CD27^+^CD38^-^ cells, plasmablasts were defined as CD45^+^CD19^+^CD27^+^CD38^+^ cells), plasma cells were defined as CD45^+^CD19^+^CD27^+^CD38^+^CD138^+^ cells), and NK cells were defined as CD45^+^CD3^-^CD16^+^CD56^+^ cells. System inflammation markers such as white blood cells (WBC), neutrophil, lymphocyte, monocyte, and platelet were collected. Additionally, the neutrophil-to-lymphocyte ratio (NLR), platelet-to-lymphocyte ratio (PLR), LMR, lymphocyte-to-monocyte ratio (LMR), and systemic immune-inflammation index (SII) were derived in accordance with their respective calculation formulas.

The follow-up started when patients received treatment at our center. We follow up with patients via phone or WeChat to obtain the MG-ADL score every month. Patients who achieve an MG-ADL score of 0 or 1 are classified as achieving minimal symptom expression (MSE). Patients come to our center every 1–3 months for re-examination and adjusting drug dosage.

### Rituximab regimes

In this cohort, all 10 (100.0%) MuSK-MG patients received RTX treatment. The low-dose RTX regimen consisted of 500 to 700 mg administered over two consecutive days (100 mg on day 1 and the remaining dose on day 2) as induction therapy, followed by maintenance treatments every 6–12 months at doses of 300 to 500 mg. However, Patient 2 and Patient 7 did not proceed with subsequent RTX cycles due to personal reasons after completing one treatment cycle.

### Statistical analysis

Statistical analyses were performed using Statistical Package for the Social Sciences (SPSS 26.0) and GraphPad Prism software (version 9.2.0). Categorical variables were presented as numbers (percentages), normally distributed variables as mean ± standard deviation (SD), and non-normally distributed variables as median (interquartile range). Independent datasets were compared using the unpaired t-test (for normally distributed data) or the Mann–Whitney U test (for non-normally distributed data). For longitudinal assessment of MG-ADL and QMG scores across baseline to 24 months, we employed linear mixed-effects models to account for within-subject correlations and handle missing data under the missing-at-random assumption. To compare the dosage of prednisone from baseline to the last visit, we employed Friedman’s test. Subsequently, *post hoc* pairwise comparisons were conducted using Dunn’s multiple comparisons test. Changes in MG-ADL and QMG scores were expressed as mean change with a 95% confidence interval (CI). A two-tailed P value of < 0.05 was considered statistically significant.

## Results

### Baseline clinical characteristics of the MuSK-MG cohort

The general clinical characteristics were presented in **Table 1**. The cohort comprised exclusively female patients, with a mean onset age of 44.3 ± 12.0 years old and a mean disease duration of 5.5 ± 3.4 months. The onset symptom presentation included bulbar muscle weakness in 9/10 (90.0%) patients, ocular muscle weakness in 7/10 (70.0%) patients, while limb weakness and respiration involvement were observed in 8/10 (80.0%) and 4/10 (40.0%) patients, respectively. In this study, the presence of respiratory muscle involvement in patients was assessed using the MG-ADL score, alongside determining whether the forced vital capacity (FVC) was < 80% of the predicted value. Prior to RTX, patients received the following therapies: pyridostigmine (8/10, 80.0%), prednisone (9/10, 90.0%), tacrolimus (4/10, 40.0%), and IVIg (1/10, 10.0%). The MGFA classification distribution before RTX was II:III:IV = 2:5:3. The baseline clinical assessment showed a mean MG-ADL score of 8.7 ± 2.5 and a mean QMG score of 13.3 ± 3.7.

**Table 1:** Basic clinical features and treatments of the MuSK-MG cohort.

No.	Gender	Age at onset, years	Duration, months	Symptoms at disease onset	MGFA classification	MG-ADL score	QMG score	Previous treatment	Dose of prednisone, mg/day	Duration of prednisone, months	Follow-up, months	Times of RTX cycles
1	Female	47	1	Ocular, Bulbar	IIIb	5	10	Py	/	/	42	4
2	Female	67	7	Bulbar, Limbs, Respiration	IVb	12	17	P, TAC	60	2	39	1
3	Female	29	3	Ocular, Limbs	IIa	6	12	Py, P, TAC	25	1	38	3
4	Female	35	10	Ocular, Limbs, Bulbar, Respiration	IIIa	8	14	Py, P, TAC	30	5	34	5
5	Female	54	3	Ocular, Limbs, Bulbar	IVb	10	12	P	60	1	30	3
6	Female	54	6	Limbs, Bulbar, Respiration	IVb	12	14	Py, P, IVIG	30	1	25	2
7	Female	45	12	Limbs, Bulbar, Respiration	IIIb	10	12	Py, P	50	1	24	1
8	Female	29	4	Ocular, Limbs, Bulbar	IIIa	7	14	Py, P	60	2	20	3
9	Female	44	3	Ocular, Limbs, Bulbar	IVb	9	13	Py, P, TAC	40	1	17	2
10	Female	39	6	Ocular, Bulbar	IIb	4	4	Py, P	60	2	12	2

GC, glucocorticoid; IVIg, intravenous immunoglobin; MG-ADL, myasthenia gravis-specific activities of daily living; MGFA, Myasthenia Gravis Foundation of America; MuSK, muscle-specific tyrosine kinase; P, prednisone; Py, pyridostigmine; QMG, quantitative myasthenia gravis; RTX, rituximab; TAC, tacrolimus.

### Comparison of clinical and immunological features between MuSK-MG and AChR-MG

**Figure 1:**
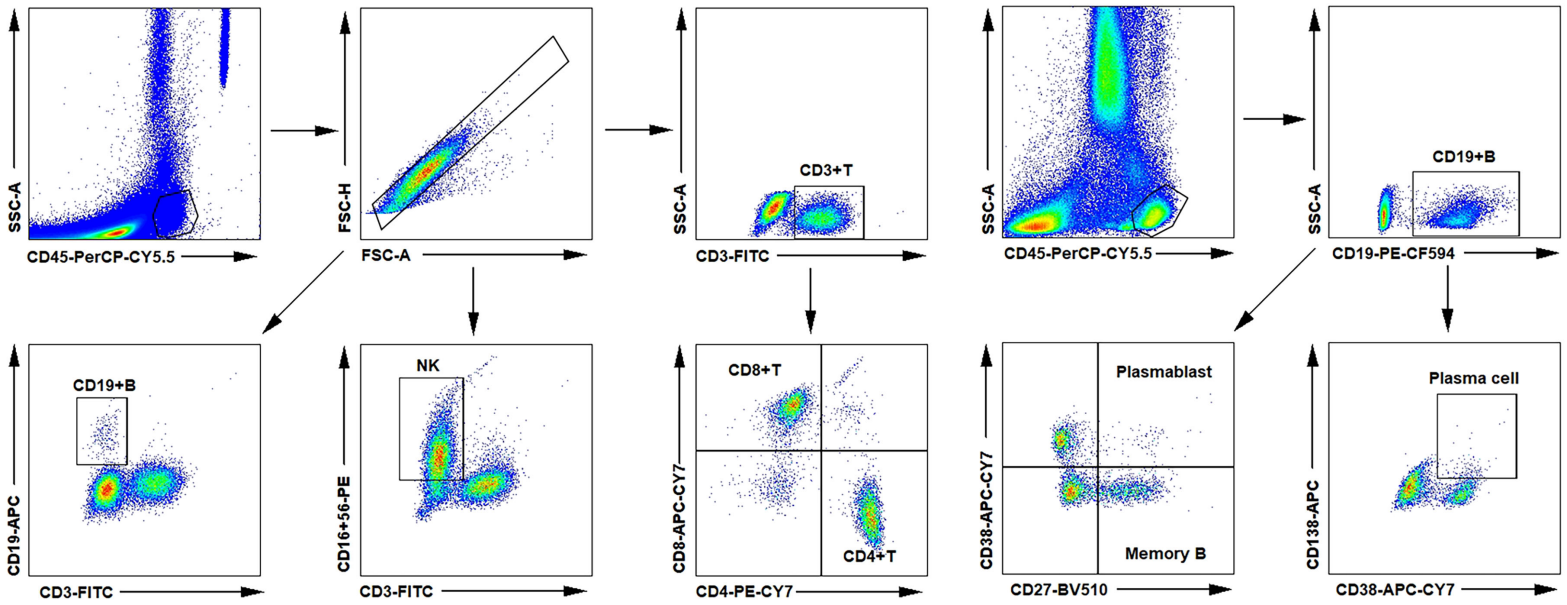
Flow cytometry analysis of the proportions of T-cell, B-cell subsets, and NK cells in circulation. AChR, acetylcholine receptor; MG, myasthenia gravis; MuSK, muscle-specific tyrosine kinase; NK, natural killer.

To investigate the clinical and immunological features of patients with MuSK-MG, we further collected 10 age- and gender-matched new-onset patients with AChR-MG. In the MuSK-MG cohort, patients 2 and 3 were not included in this part of the analysis due to incomplete immunological records. The general characteristics of two groups were presented in **Table 2**. No significant difference was detected in gender, age, duration of disease, and baseline MGFA classification between two groups (all P > 0.05, **Table 2**). Comparing the distribution of symptoms at disease onset between two groups, we found that the proportion of bulbar muscle weakness in MuSK-MG was significantly higher than AChR-MG (P = 0.036, **Table 2**). The QMG score and MG-ADL score in those MuSK-MG patients were 13.0 ± 3.9 and 8.6 ± 2.3, which were higher than AChR-MG patients (both P < 0.05, **Table 2**). The proportions of T-cell subsets, B-cell subsets, and NK cells were compared between the two groups. The results of flow cytometry analysis regarding the proportions of these cell types (T-cell subsets, B-cell subsets, and NK cells) are presented in **Figure 1**. We found that the proportions of CD19^+^B cells and memory B cells were higher in MuSK-MG patients than in AChR-MG patients (P < 0.001; P = 0.003, **Table 2**). No significant difference was detected in CD3^+^T cells, CD4^+^T cells, CD8^+^T cells, NK cells, plasmablasts, and plasma cells (all P > 0.05, **Table 2**). In addition, there was no difference in WBC, neutrophil, lymphocyte, monocyte, platelet, NLR, PLR, LMR, and SII (**Table 2**).

**Table 2:** Comparison of the clinical and immunological characteristics between MuSK-MG patients and new-onset AChR-MG patients.

Variables	MuSK-MG (n = 8)	AChR-MG (n = 10)	t/z/χ^2^	P-value
Female, n (%)	8 (100)	10 (100)	–	–
Age, years	43.4 ± 8.8	49.2 ± 14.2	-0.996	0.334
Duration, months	5.0 (3.0, 9.0)	2.2 (1.7, 7.5)	0.987	0.360
GMG, n (%)	8 (100.0)	10 (100)	–	–
MGFA classification				
II: III : IV, n	1:5:2	4:2:2	1.717	0.482
Muscle group distribution at disease onset, n (%)				
Ocular	6 (75.0)	9 (90.0)	0.720	0.412
Limbs	6 (75.0)	9 (90.0)	0.720	0.559
Bulbar	8 (100.0)	5 (50.0)	5.538	0.036
Respiration	3 (37.5)	3 (30.0)	0.112	1.000
MG-ADL score	8.6 ± 2.3	5.9 ± 2.7	2.267	0.038
QMG score	13.0 ± 3.9	8.6 ± 2.3	2.348	0.032
Previous treatments				
Pyridostigmine, n (%)	7 (87.5)	–	–	–
Prednisone, n (%)	7 (87.5)	–	–	–
Tacrolimus, n (%)	2 (25.0)	–	–	–
IVIg, n (%)	1 (12.5)	–	–	–
CD3^+^T cells (%)	65.7 ± 7.1	69.2 ± 13.9	-0.645	0.528
CD4^+^T cells (%)	42.8 (29.9, 47.0)	43.1 (32.3, 46.2)	-0.089	1.000
CD8^+^T cells (%)	19.2 ± 6.1	25.7 ± 9.1	-1.706	0.107
CD19^+^B cells (%)	18.8 ± 2.3	11.5 ± 3.9	4.640	<0.001
NK cells (%)	14.4 ± 8.2	18.2 ± 12.6	-0.731	0.476
Memory B cells (%)	0.459 (0.323, 1.226)	0.198 (0.076, 0.283)	2.843	0.003
Plasmablasts (%)	0.026 (0.013, 0.064)	0.028 (0.021, 0.032)	0.089	0.965
Plasma cells (%)	0.013 ± 0.009	0.012 ± 0.009	0.451	0.658
White blood cell (10^9^/L)	7.9 ± 3.1	6.4 ± 1.5	1.306	0.222
Neutrophil (10^9^/L)	5.0 ± 2.3	3.9 ± 1.1	1.373	0.189
Lymphocyte (10^9^/L)	2.3 ± 1.0	1.9 ± 0.4	1.001	0.344
Monocyte (10^9^/L)	0.4 ± 0.2	0.4 ± 0.2	0.689	0.501
Platelet (10^9^/L)	267.1 ± 58.7	224.5 ± 62.4	1.478	0.159
NLR	2.3 ± 1.0	2.0 ± 0.4	0.835	0.425
PLR	133.6 ± 42.2	118.0 ± 34.5	0.667	0.514
LMR	5.7 ± 2.3	5.5 ± 1.5	0.267	0.793
SII	604.8 ± 234.5	441.8 ± 115.7	1.934	0.071

AChR, acetylcholine receptor; GMG, general myasthenia gravis; IVIg, intravenous immunoglobin; LMR, lymphocyte-to-monocyte ratio; MG-ADL, myasthenia gravis-specific activities of daily living; MGFA, Myasthenia Gravis Foundation of America; MuSK, muscle-specific tyrosine kinase; NK, natural killer; NLR, neutrophil-to-lymphocyte ratio; PLR, platelet-to-lymphocyte ratio; QMG, quantitative myasthenia gravis; SII, systemic immune-inflammation index.

### Correlation between peripheral lymphocyte proportions and disease severity in MuSK-MG

**Figure 2:**
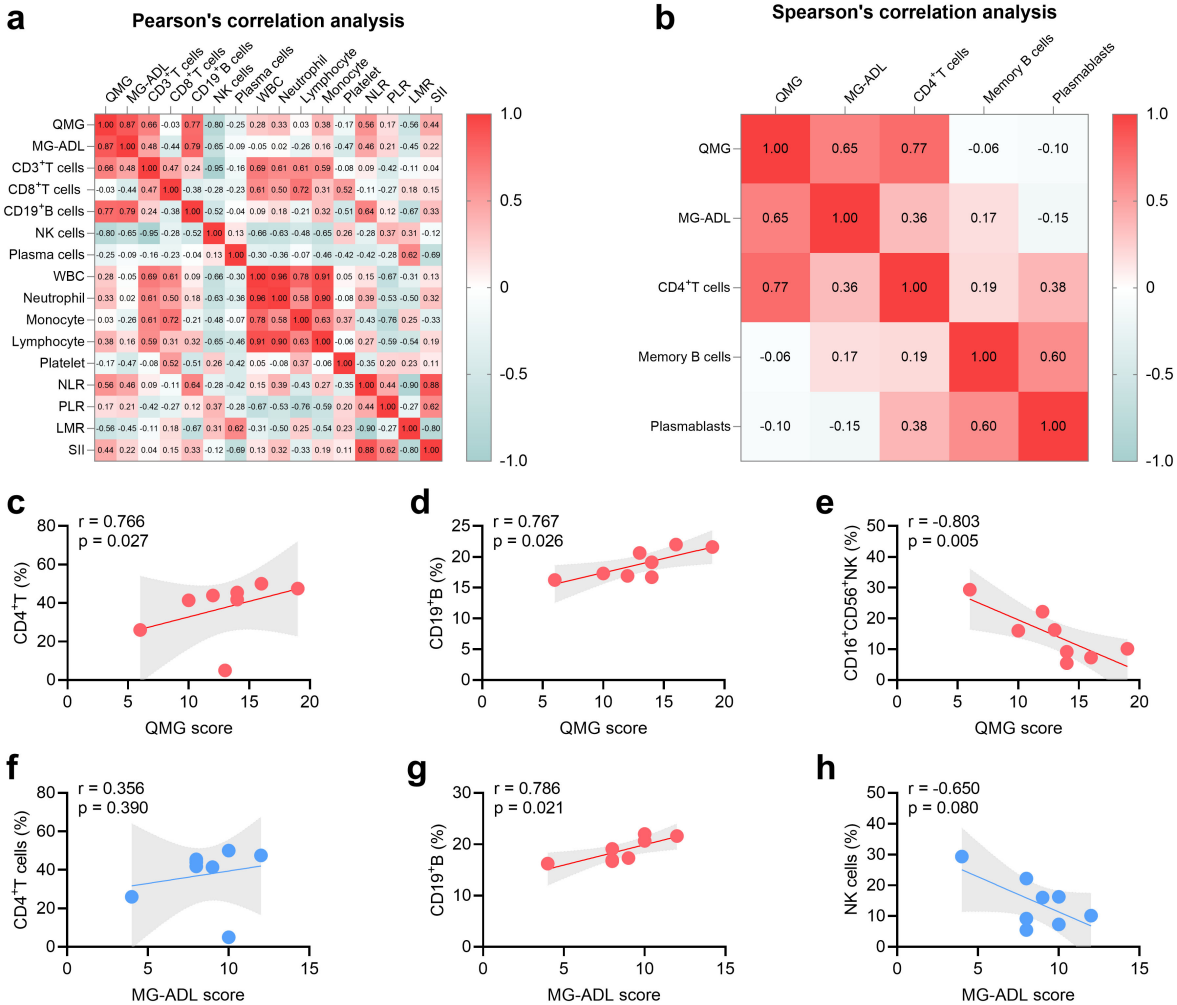
Relationship between severity of MuSK-MG (n = 8) and T-cell subsets, B-cell subsets, NK cells, and system inflammation markers. **(a, b)** Heat map displaying correlations of severity of MuSK-MG (n = 8) with T-cell subsets, B-cell subsets, NK cells, and system inflammation markers (Pearson’s correlation analysis was utilized in panel **(a)** and Spearman’s correlation analysis was utilized in panel **b**). **(c-e)** Correlation analysis of QMG score with CD4^+^T, CD19^+^B, and NK cells. **(f-h)** Correlation analysis of MG-ADL score with CD4^+^T, CD19^+^B, and NK cells. A two-tailed P value of < 0.05 was considered statistically significant. LMR, lymphocyte-to-monocyte ratio; MG-ADL, myasthenia gravis-specific activities of daily living; NK, natural killer; NLR, neutrophil-to-lymphocyte ratio; PLR, platelet-to-lymphocyte ratio; QMG, quantitative myasthenia gravis; SII, systemic immune-inflammation index; WBC, white blood cell.

The relationship between peripheral lymphocyte proportions, inflammatory index, and disease severity before RTX treatment was shown as heatmaps in **Figures 2a, b**. Spearman’s correlation analysis demonstrated that the proportion of CD4^+^T cells was positively related to QMG score (r = 0.766, P = 0.027, **Figure 2c**) and the proportion of NK cells was negatively related to MG-ADL score (r = -0.803, P = 0.005, **Figure 2e**). However, no relation was detected between MG-ADL score and CD4+T cells and NK cells (**Figures 2f, h**). Furthermore, Pearson’s correlation analysis demonstrated that the proportion of CD19^+^B cells was positively related to QMG score (r = 0.767, P = 0.026, **Figure 2d**) and MG-ADL score (r = 0.786, P = 0.021, **Figure 2g**) in MuSK-MG. No statistical relationship between WBC, neutrophil, lymphocyte, monocyte, platelet, NLR, PLR, LMR, SII, and severity of MG was found in this MuSK-MG cohort (**Figures 2a, b**).

### Efficacy of rituximab in MuSK-MG

**Figure 3:**
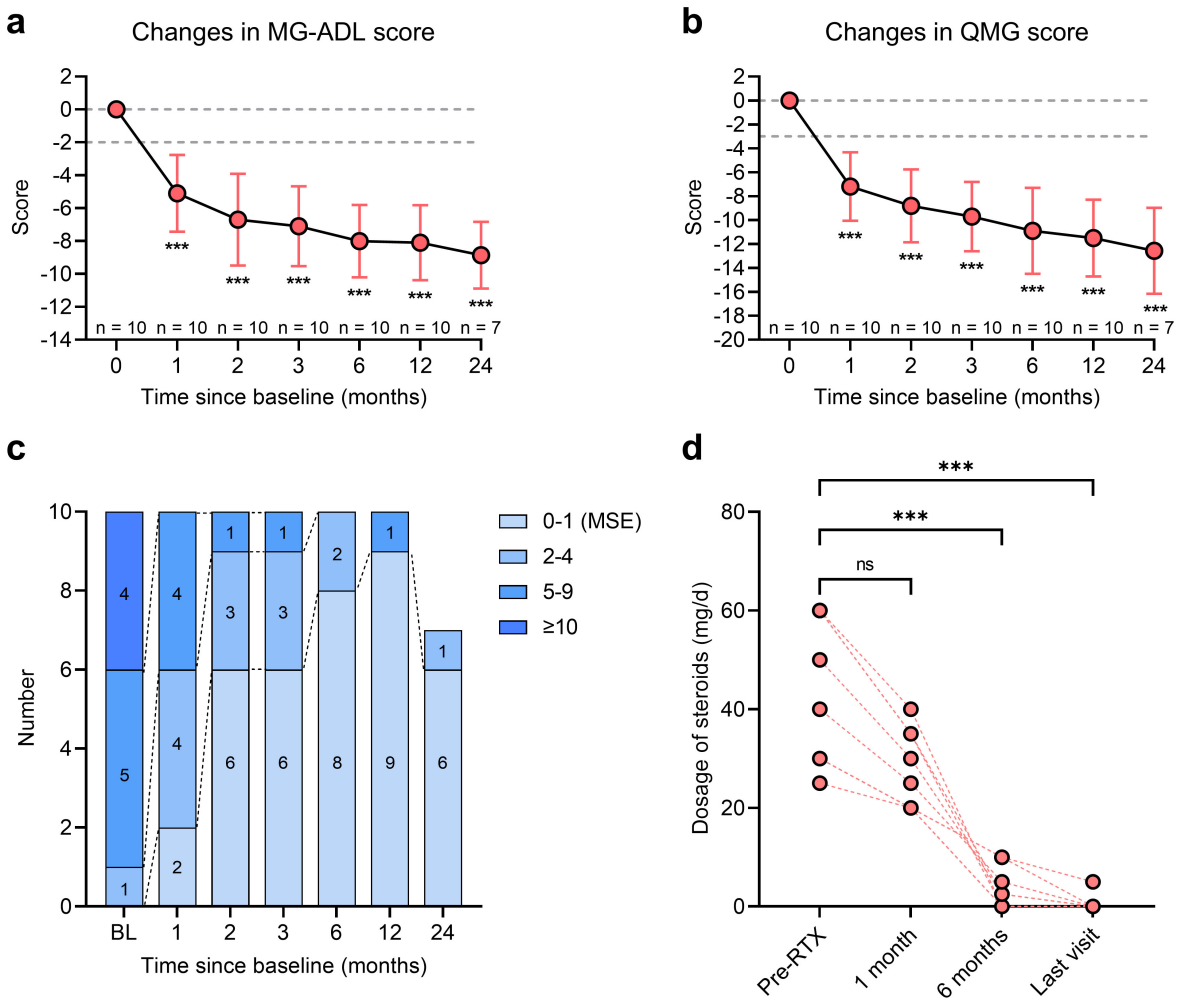
Clinical improvement of MuSK-MG (n = 10) after RTX treatment. **(a, b)** MG-ADL score and QMG score changes from baseline to month 24 after RTX treatment; **(c)** The stratification of MuSK-MG from baseline to month 24 after RTX treatment; **(d)** Changes in dosage of prednisone from baseline to the last visit. ***P < 0.001, ns: no significance. MG, myasthenia gravis; MG-ADL, myasthenia gravis-specific activities of daily living scale; MuSK, muscle-specific tyrosine kinase; QMG, quantitative myasthenia gravis; RTX, rituximab.

The changes in MG-ADL and QMG scores following RTX treatment are summarized in **Table 3**. At one-month post-RTX, 9 of 10 patients (90.0%) achieved clinically meaningful improvement (CMI), defined as a ≥2-point reduction in MG-ADL score. The mean MG-ADL score significantly decreased from 8.7 ± 2.5 to 3.6 ± 2.3 at month 1 (mean reduction: -5.1 [95% CI: -7.6 to -2.6]; P < 0.001; **Figure 3a**). The mean QMG score declined from 13.3 ± 3.7 to 6.1 ± 2.3 (mean reduction: -7.2 [95% CI: -10.2 to -4.2]; P < 0.001; **Figure 3b**). From month 2 to month 24, all 10 patients (100.0%) attained CMI. By month 24, the mean reductions in MG-ADL and QMG scores were -8.0 (95% CI: -11.0 to -5.0) and -11.4 (95% CI: -16.6 to -6.3), respectively (both P < 0.001; **Figures 3a, b**).

**Table 3:** Changes in MG-ADL, QMG scores, and daily dose of prednisone in MuSK-MG patients after rituximab treatment.

Variables	Baseline (n = 10)	1 month (n = 10)	2 months (n = 10)	3 months (n = 10)	6 months (n = 10)	12 months (n = 10)	24 months (n = 7)
MG-ADL score	8.7 ± 2.5	3.6 ± 2.3	2.0 ± 1.9	1.6 ± 1.7	0.5 (0.0, 1.2)	0.0 (0.0, 0.2)	0.0 (0.0, 1.0)
QMG score	13.3 ± 3.7	6.1 ± 2.3	4.5 ± 2.5	3.6 ± 1.9	2.4 ± 1.9	1.8 ± 2.0	1.8 ± 2.3
Daily dose of prednisone, mg	40.0 (30.0, 60.0)	25.0 (20.0, 35.0)	20.0 (15.0, 22.5)	10.0 (10.0, 20.0)	2.5 (0.0, 7.5)	–	–

MG, myasthenia gravis; MG-ADL, myasthenia gravis-specific activities of daily living scale; MuSK, muscle-specific kinase; n, number; QMG, quantitative myasthenia gravis.

Patients were stratified by clinical severity according to MG-ADL scores from baseline to month 24. The proportion of patients with severe symptoms (MG-ADL score >10) significantly declined from 40.0% (n = 4) at baseline to 0% by month 1 (**Figure 3c**). The number of patients with moderate clinical activity (MG-ADL score 5-9) decreased from 50.0% (n = 5) at baseline to 10.0% (n = 1) at month 2 and further to 0% by month 24 (**Figure 3c**). The proportion of patients achieving MSE rose from 0% at baseline to 20.0% (n = 2) at month 1, 90.0% (n = 9) at month 12, and 85.7% (n = 6) at month 24 (**Figure 3c**). No patient-reported adverse event, such as infections and allergic reactions, was reported in all patients.

### Steroid−sparing effect of rituximab

In this study, 9/10 (90.0%) patients had received steroids prior to RTX, with a median prednisone dosage of 40.0 (30.0, 60.0) mg/day (**Table 3**). The changes in daily dosage of prednisone were presented in **Table 3**. By month 1, the median prednisone dosage decreased to 25.0 (20.0, 35.0) mg/day and further decreased to 2.5 (0.0, 7.5) mg at month 6 (**Figure 3d**, P < 0.001). From 12 months after RTX to the last follow-up visit, 8/9 (88.9%) patients had discontinued oral prednisone, while the remaining patient was maintained with a low dose of 5 mg/day.

## Discussion

In this study, we investigated the clinical features and immunological profiles of MuSK-MG patients and observed the efficacy of RTX treatment. All patients in our study were female, showing a higher prevalence, consistent with previous studies (20–22). The possible mechanism is that human immune cell composition varies by sex, and estrogen and androgens have different regulatory effects on T and B cells (21, 23, 24). For example, the proportions of CD4^+^T cells, CD19^+^B cells, and plasma cells are higher in females (25–27). Also, androgens inhibit B cell lymphopoiesis, affect B cell progenitors, and upregulate the expression of B cell activating factor (BAFF) crucial for B cell development and maturation (28, 29). However, the mechanisms underlying the female predominance in MuSK-MG pathogenesis require further investigation. In our study, the most common muscle group disease was bulbar muscle, significantly higher than AChR-MG. Previous studies showed that over 80% of MuSK-MG patients had bulbar muscle weakness (21, 30, 31). The susceptibility of bulbar muscle also implies that MuSK-MG patients are more likely to experience acute exacerbations and even myasthenic crisis (5).

MuSK is a receptor tyrosine kinase located at the NMJ and plays a vital role in coordinating acetylcholine receptor aggregation and maintaining the structural integrity of the postsynaptic apparatus (14). Unlike anti-AChR or anti-LRP4 antibodies, MuSK-MG pathology is mainly mediated by IgG4 subclass antibody from short-lived plasmablasts (SLPBs). The immune imbalance and abnormal activation of T-cell and B-cell subsets are the fundamental mechanism of MG related pathogenic antibodies production. In this study, we found that MuSK-MG patients had higher proportions of CD19^+^B cells and memory B cells, suggesting that patients with MuSK-MG suffer from a higher B cells load. Besides, we also found a positive relationship between CD19^+^B cells and severity of MG. This result also demonstrates to some extend why anti-CD20 agents are particularly effective in improving symptoms in MuSK-MG patients, although the more important reason is that anti-MuSK antibodies are mainly produced by SLPBs (expressing CD20) (12, 32). In addition, we observed a correlation between MG activity and proportions of CD4^+^T cells. A previous study has demonstrated that CD4^+^T cells from MuSK-MG patients more frequently produced interleukin (IL)-2, TNF-α, and IL-17 (33). This may suggest that although the expression levels of CD4^+^T cells in MuSK-MG patients are normal, the function of these cells may be abnormally activated. NK cells are an important element of innate immunity and participate in the pathogenesis of MG (34, 35). Zhang et al. found total NK cell frequency was lower in MGFA II-IV patients than in healthy controls and MGFA I patients (34). This suggested a negative correlation between NK cell frequency and MG activity. Our study also found a negative correlation between NK cells and QMG score in MuSK-MG. Nevertheless, considering that this is a small-sample retrospective investigation and the patients had undergone immunotherapy previously, which probably affected the immunological characteristics of MuSK-MG—for instance, exogenous glucocorticoids can trigger classical T-cell apoptosis—these findings necessitate further verification.

RTX is a therapy for B-cell depletion, which targets CD20-positive cells such as pre-B cells, immature B cells, and plasmablasts. Since SLPBs express CD20 and mainly generate anti-MuSK antibodies, RTX can efficiently ameliorate the symptoms of MuSK-MG and lower the titers of anti-MuSK antibodies (13, 36). According to a latest meta-analysis which included 111 MuSK-MG patients, 82% of patients could achieve minimal manifestations or better status after RTX (37). In this study, all the patients under observation attained CMI. Besides, 90% of the patients reached the MSE status 12 months after RTX treatment. However, more studies are needed to explore how often RTX is administered, and what dose of RTX is effective in depleting B cells and maintaining minimal manifestations in MuSK-MG patients.

Our study has some limitations. Firstly, the relatively small sample size might potentially constrain the statistical power and the generalizability of our research findings. Future studies with a larger sample size are essential to further validate our results. Second, without a control group with MuSK-MG receiving alternative therapies or no RTX, we cannot definitively attribute clinical effects to RTX alone. Third, pre-existing immunosuppressants may have influenced immune profiles and treatment, while this reflects real-world practice but also was a potential confounder. Additionally, the lack of functional assessments (e.g., cytokine profiles, T-cell markers) limits our ability to correlate cellular phenotypes with clinical relevance. Future studies should incorporate treatment-naïve cohorts, standardized washout periods, and functional immune assays.

## Conclusion

In conclusion, this study preliminarily explored the distinct immunological characteristics of MuSK-MG and further detected the efficacy of RTX in inducing sustained clinical improvement. Despite the existing limitations, our findings add to the increasing evidence supporting the use of RTX in MuSK-MG and lay a foundation for future research aimed at optimizing treatment strategies and enhancing patient outcomes.

## Data Availability

The original contributions presented in the study are included in the article/Supplementary Material. Further inquiries can be directed to the corresponding author/s.
